# Gamete-exporting organs of vertebrates: dazed and confused

**DOI:** 10.3389/fcell.2023.1328024

**Published:** 2023-12-22

**Authors:** Akira Kanamori, Yasuhisa Kobayashi

**Affiliations:** ^1^ Group of Development and Growth Regulation, Division of Biological Science, Graduate School of Science, Nagoya University, Nagoya, Japan; ^2^ Laboratory for Aquatic Biology, Department of Fisheries, Faculty of Agriculture, Kindai University, Nara, Japan

**Keywords:** Müllerian duct, oviduct, Wolffian duct, sperm duct, *wnt4*, teleosts, cartilaginous fishes, cyclostomes

## Abstract

Mature gametes are transported externally for fertilization. In vertebrates, the gonads are located within the coelom. Consequently, each species has specific organs for export, which often vary according to sex. In most vertebrates, sperm ducts and oviducts develop from the Wolffian and Müllerian ducts, respectively. However, exceptions exist. Both sexes of cyclostomes, as well as females of basal teleosts, lack genital ducts but possess genital pores. In teleosts of both sexes, genital ducts are formed through the posterior extensions of gonads. These structures appear to be independent of both Wolffian and Müllerian ducts. Furthermore, the development of Wolffian and Müllerian ducts differs significantly among various vertebrates. Are these gamete-exporting organs homologous or not? A question extensively debated around the turn of the 20th century but now largely overlooked. Recent research has revealed the indispensable role of Wnt4a in genital duct development in both sexes of teleosts: zebrafish and medaka. *wnt4a* is an ortholog of mammalian *Wnt4*, which has functions in Müllerian duct formation. These results suggest a potential homology between the mammalian Müllerian ducts and genital ducts in teleosts. To investigate the homology of gamete-exporting organs in vertebrates, more detailed descriptions of their development across vertebrates, using modern cellular and genetic tools, are needed. Therefore, this review summarizes existing knowledge and unresolved questions on the structure and development of gamete-exporting organs in diverse vertebrate groups. This also underscores the need for comprehensive studies, particularly on cyclostomes, cartilaginous fishes, basal ray-finned fishes, and teleosts.

## 1 Introduction

The gonads of all vertebrates are suspended dorsally within the coelom (body cavity). Consequently, for sexual reproduction to occur, gametes must find their way out of the body. Vertebrates employ various paths for this purpose, often exhibiting differences based on sex. Some have genital pores, which are very short passages from the coelom to the urogenital sinus. Most jawed vertebrates use the Wolffian ducts (WDs) and Müllerian ducts (MDs) for sperm and ova export, respectively. Finally, most teleosts of both sexes have genital ducts that develop as posterior extensions of the gonads. The anatomical differences among these three types of structures have historically led to their classification as non-homologous organs in textbooks on vertebrate comparative anatomy (e.g., Romer and Parsons, 1977; Wake, 1979; Blüm, 1986; Lombardi, 1998).

Recent studies have revealed that Wnt4a is indispensable for genital duct development in both sexes of two diverse teleost species, zebrafish (Kossack et al., 2019) and the medaka *Oryzias latipes* (Kanamori et al., 2023). Teleost *wnt4a* is an ortholog of mammalian *Wnt4*, which has functions in the MD development (mice, Vainio et al., 1999; humans, Biason-Lauber et al., 2004, Biason-Lauber et al., 2007; see also Mullen and Behringer, 2014; Gonzales et al., 2021). These findings suggested the existence of homologous processes during the development of mammalian MDs and genital ducts in teleosts. This proposition prompted us to question whether homology exists among all gamete-exporting organs across vertebrates. During our literature searches, we were intrigued to find that this specific subject was among the favorites of comparative anatomists around the turn of the 20th century. Their curiosity was likely stimulated by the remarkable diversity observed in both the anatomy and developmental modes of gamete-exporting organs. Darwinian evolutionary theory and Mendelian genetics, combined with advances in microscopy, led to enthusiastic quests for homologies among morphological diversities so prevalent in the animal kingdom. Hoar (1969) noted: “The comparative anatomist has found some of his most interesting problems in the phylogeny of the gonoducts [genital ducts; square brackets represent the authors’ annotations] and their relationships to the mesonephric tubules and ducts.” Kerr (1919) and Goodrich (1930) provided summaries of the literature up to this period. Particularly, Edwin S. Goodrich made significant contributions to the discussion of genital duct evolution in the animal kingdom, including vertebrates. His enduring interest in the subject is exemplified by a trilogy of papers (Goodrich, 1895; Goodrich, 1930; Goodrich, 1945; see also Holland, 2017). The most debated topics at the time were 1) the origin of genital pores in cyclostomes, 2) the two distinct modes of MD development (cartilaginous fishes vs. other jawed vertebrates), and 3) the origin of teleost genital ducts. Lo and behold, even after a century, these questions remain largely unanswered, with research on the subject tapering off since the 1940s. The sole exception in recent times has been Karl-Heinz Wrobel, who authored a series of exquisite papers on the anatomy and development of the urogenital system in sturgeons during the last few years of his career (Wrobel and Süß, 2000; Wrobel et al., 2002a; Wrobel et al., 2002b; Wrobel, 2003; Wrobel and Jouma, 2004). We believe that these studies provide a roadmap for future investigations, emphasizing the need for detailed and precise descriptions of the structure and development of gamete-exporting organs in several key groups of vertebrates.

How did these seemingly distinct structures, crucial for avoiding extinction, differentiate over the course of vertebrate evolution? This review aims to rekindle general interest in this intriguing, long-unanswered problem. It starts with 1) a summary of the knowledge on vertebrate gamete-exporting organs, followed by 2) detailed discussions addressing pertinent unanswered questions. Finally, we propose 3) the latest methodologies, in addition to classical histology, to address these questions.

## 2 Anatomy and phylogeny of gamete-exporting organs in vertebrates

**FIGURE 1:**
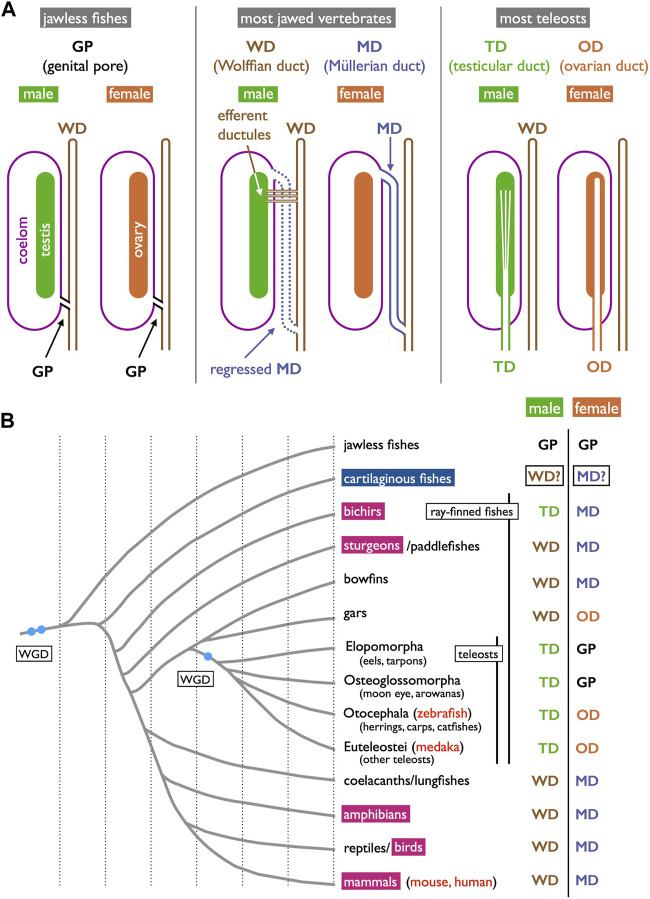
Three types of gamete-exporting organs in vertebrates. **(A)** Diagrams illustrating gamete-exporting organs in vertebrates. In jawless fishes, genital ducts are absent, and both sperm and ova are released into the coelom and exported through the genital pores (GPs). In most jawed vertebrates, sperm and ova are exported through the Wolffian ducts (WDs) and Müllerian ducts (MDs), respectively. In males, sperm are exported through the efferent ductules to the WDs; the MDs are developed but later regress in many cases. In females, mature oocytes are shed into the coelom and then exported through the opening of the MDs to the urinary sinus. Finally, most teleosts develop genital ducts as the posterior elongation of gonads: testicular ducts (TDs) and ovarian ducts (ODs). **(B)** Phylogenetic distribution of gamete-exporting organs. The phylogeny of vertebrate groups is based on Hughes et al. (2018), Tekezaki (2021), Koepfli et al. (2015), and Holland and Ocampo Daza (2018). In the species indicated in red letters, *Wnt4* orthologs have been reported to have functions in genital duct development. The **invagination-elongation** mode of MD development is observed in the groups marked in carmine red squares. In cartilaginous fishes (cerulean blue square), the MDs (and the WDs) develop through the **longitudinal splitting** of the pronephric ducts (see Section 3.4 for details).

As previously outlined, we have broadly categorized the gamete-exporting organs in vertebrates into three main types: genital pores, WDs and MDs, and teleost-specific genital ducts (Figure 1A). Several variations exist within each type, which are not discussed in detail in this review. Interested readers seeking more comprehensive information may refer to textbooks by Goodrich (1930), Romer and Parsons (1977), Wake (1979), Blüm (1986), and Lombardi (1998). Goodrich’s work is the oldest and covers a wide array of vertebrate genital duct anatomy and development. The other four offer similar coverage but with the distinctive viewpoints of their respective authors. Below, we provide a summary of the accumulated knowledge on the structures of gamete-exporting organs, along with their distribution within the vertebrate phylogeny.

### 2.1 Genital pores

**FIGURE 2:**
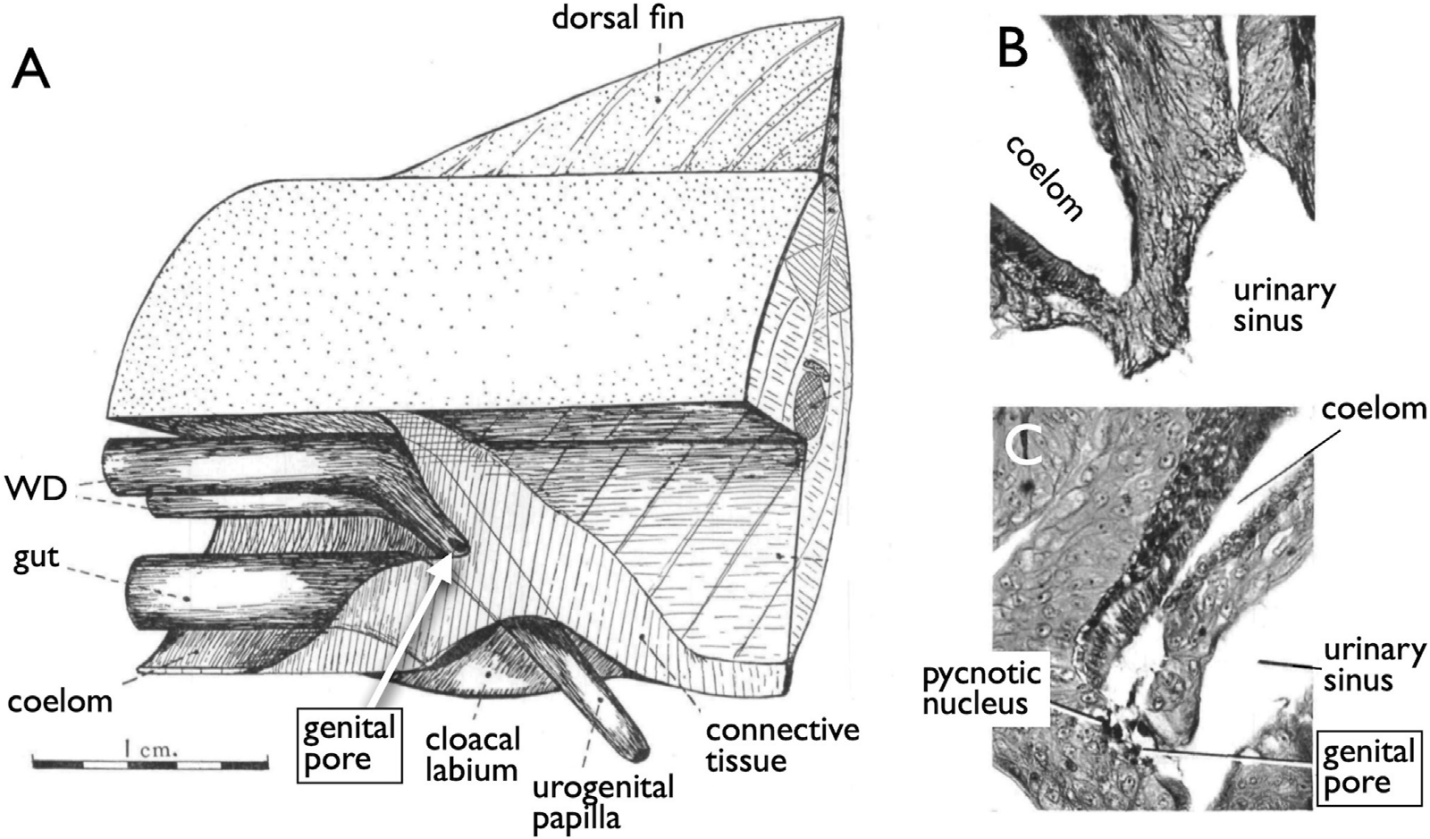
Induction of the genital pores in river lamprey by gonadotropins [reproduced from Knowles (1939), with permission from The Company of Biologists]. **(A)** View of the cloacal region of a matured adult *Lampetra fluviatilis*, which has been dissected from the left side. **(B)** Section through the cloacal region of a normal adult male. **(C)** Section through the cloacal region of an adult female, which had been injected with gonadotropins. Several cell layers between the coelom and the urinary sinus undergo apoptosis, forming a path (genital pore) for the oocytes ovulated into the coelom.

Jawless fishes, the earliest vertebrate lineage, include only two extant groups: lampreys and hagfishes (collectively known as cyclostomes). These species lack genital ducts, with mature sperm and oocytes released into the coelom and subsequently exported through genital pores (GPs). These GPs appear only when the individuals have reached maturity and are prepared for copulation. The most recent and informative literature on this topic dates back to Knowles (1939) who demonstrated that gonadotropins and steroid hormones induce GPs from the coelom to the urinary sinus in river lamprey (Figure 2). This hormonal induction led to apoptosis in several cell layers including the coelomic epithelium, mesenchyme, and nephric duct epithelium, resulting in the establishment of GPs (Figure 2B, control; Figure 2C, treated with gonadotropins).

Furthermore, it is worth noting that many basal teleosts, such as elopomorphs (eels and tarpons) and osteoglossomorphs (moon eyes and arowanas), do not possess oviducts in females (Figure 1B). In these species, mature oocytes are directly ovulated into the coelom and, similar to cyclostomes, are exported through the GPs into the urinary sinus. Descriptions of GP structures are available for eels (Syrski, 1876; Tesch, 1977) and have been briefly mentioned for moray eels (Fishelson, 1992), mooneye (*Hiodon tergisus*, Katechis et al., 2007), and pirarucu (Godinho et al., 2005). Unfortunately, comprehensive literature describing the detailed cellular structures or developmental processes of GPs in female basal teleosts is currently lacking.

### 2.2 WDs and MDs

**FIGURE 3:**
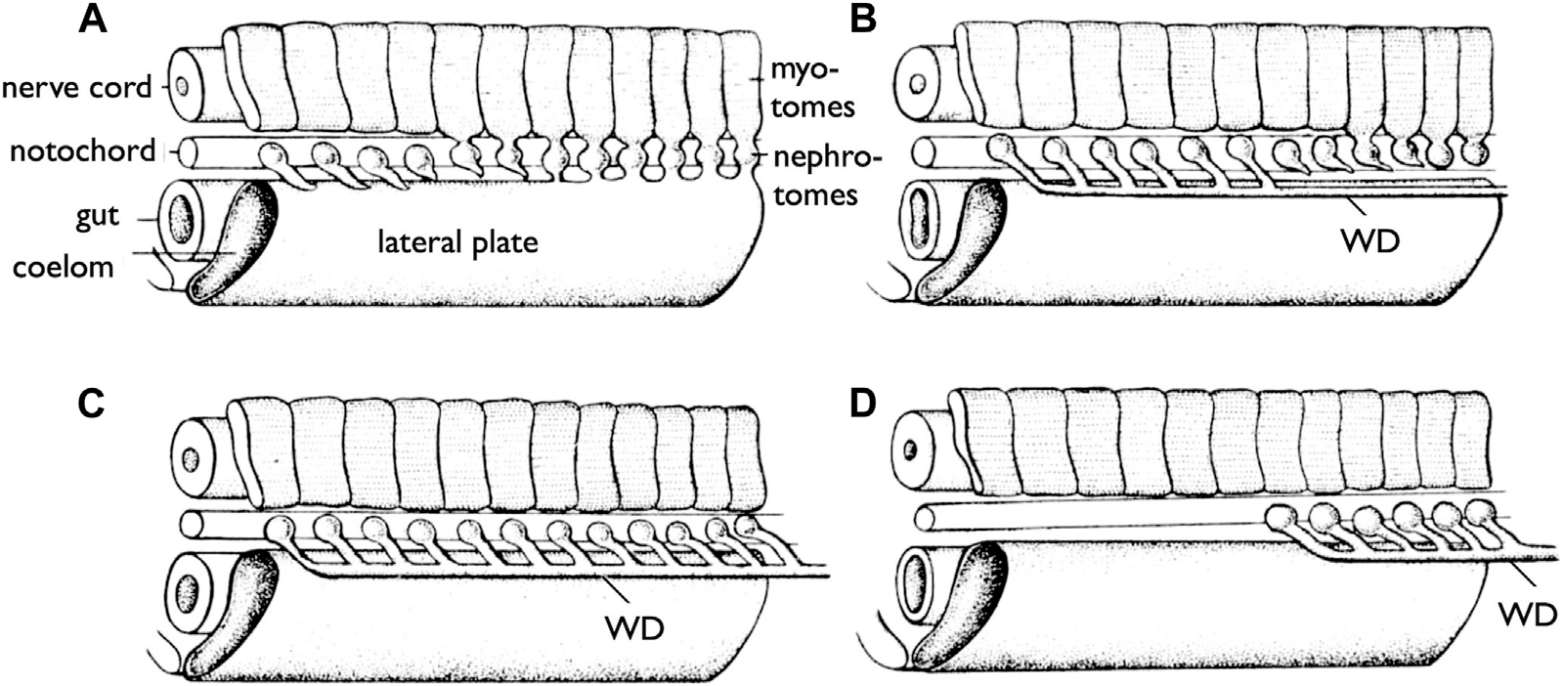
Development of the WDs [reproduced from Romer and Parsons (1977), with permission from Elsevier]. Diagrams of the anterior part of the trunk of an embryo (skin removed) to show the development of the WD. **(A)** Most anterior pronephric nephrotomes are budding out tubules that tend to fuse posteriorly. **(B)** The pronephric tubules have formed the duct (WD); the nephrotomes farther posteriorly are forming tubules which are to enter the duct (WD). **(C)** The more posterior tubules have joined the duct (WD). **(D)** The pronephros are lost, but the WD formed by it persists to drain the more posterior part of the kidney (mesonephros).

**FIGURE 4:**
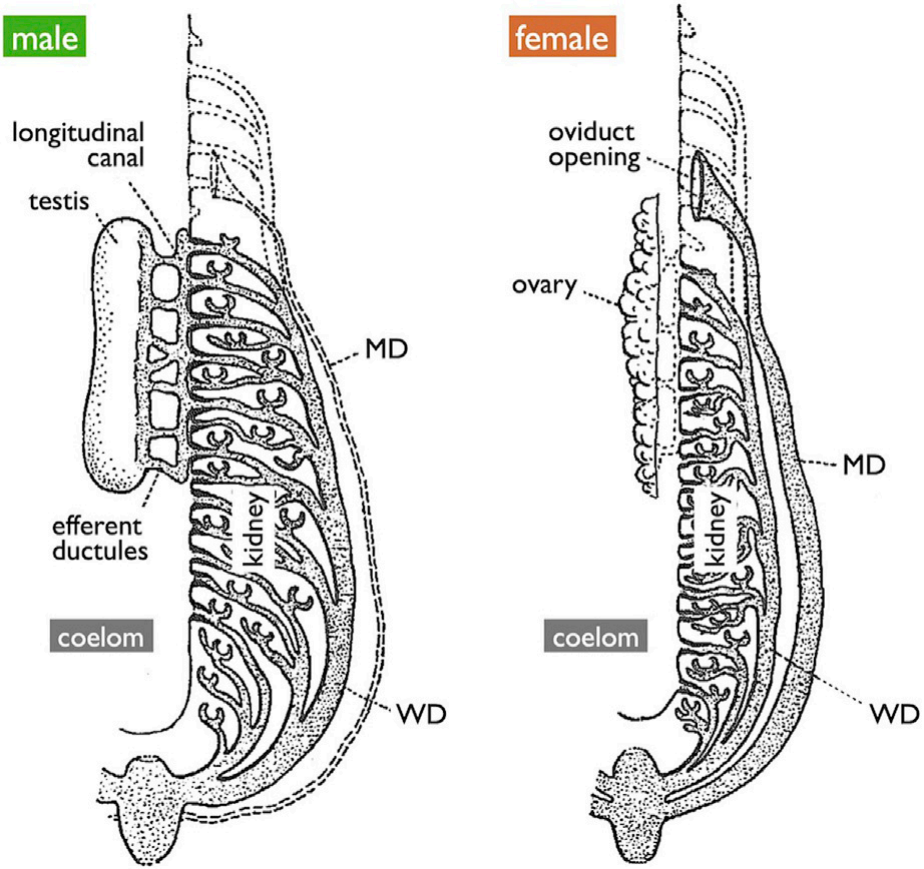
Diagrams of the urogenital system of generalized jawed vertebrates [adapted from Goodrich (1945), with permission from The Company of Biologists]. The testis is connected to the kidney through efferent ductules, and subsequently to WDs. Mature oocytes are released into the coelom and expelled through the openings of MDs. Vestigial MDs in males, vestigial networks of efferent ductules in females, and embryonic pronephros in both sexes are depicted by the dotted lines.

It is essential to clearly define WD at the outset. During the early stages of development, all vertebrate embryos develop kidneys that facilitate the removal of waste products in the form of urine. The earliest form of the kidney is known as the pronephros, which comprises a few anterior nephrons connected to a duct that extends posteriorly to the urinary sinus (Figure 3; Romer and Parsons, 1977; de Bakker et al., 2019). This duct has been referred to by various names, including the pronephric duct, archinephric duct, and WD (as per Romer and Parsons, 1977). As development progresses, the posterior nephrons of the mesonephros (or opisthonephros, posterior kidney) become functional, and the WD is once again utilized to drain urine from the mesonephros, sometimes referred to as the mesonephric duct but identical to the WD. In all male jawed vertebrates, except teleosts and bichirs (Figure 1B), the WD, or at least a portion of the WD, is employed as the duct for exporting sperm. Typically, several thin ducts develop between the testes and WD, serving as sperm canals that transport sperm from the testes to the WDs, referred to as efferent ductules (vasa efferentia) (Figure 4).

In females, all jawed vertebrates, excluding teleosts and gars (Figure 1B), utilize the MDs, also known as paramesonephric ducts, to export ova. In this review, we defined the MD purely anatomically as a duct that opens anteriorly to the coelom, runs alongside the WD, and ultimately opens posteriorly to the urinary sinus (Figure 1A and Figure 4). Mature oocytes are released into the coelom, transferred to the MD through the coelomic opening, and exported to the urinary sinus. MDs differentiate into various regions, including the uterus that support fetuses in viviparous cartilaginous fishes and mammals. In other groups, they play a role in the production of egg envelopes and other supporting materials for oocytes (see Major et al., 2022). In Section 3.4, we discuss two different modes of MD development reported in vertebrates.

### 2.3 Genital ducts in teleosts

**FIGURE 5:**
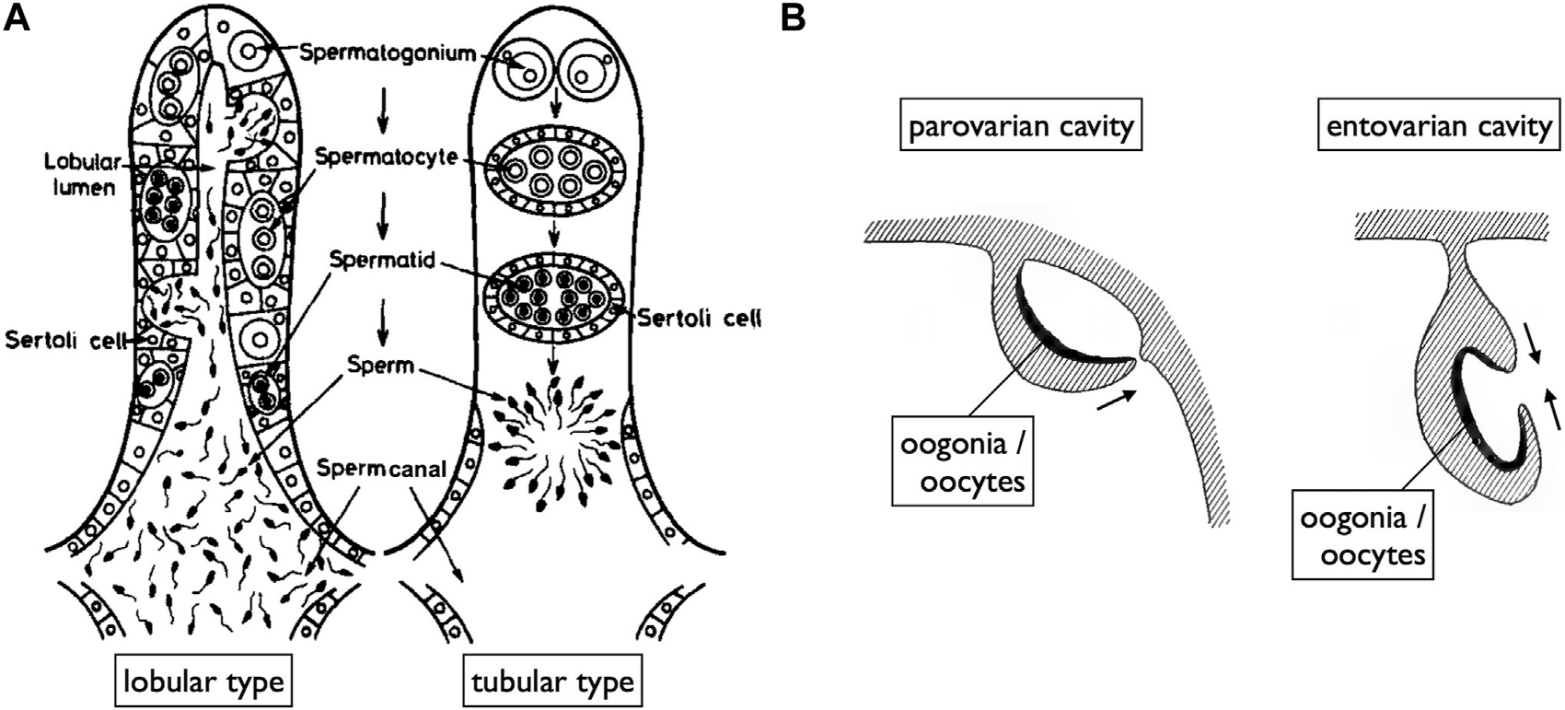
Diagrams of teleost gonads illustrating spaces for the release of mature gametes. **(A)** In both lobular type (spermatogonia distributed at various locations along spermatogenic tubes) and tubular type testes (spermatogonia localized at the tips of tubes), spermatogenesis occurs within cysts surrounded by Sertoli cells. Mature sperm are released into spaces (sperm canals) covered by epithelial cells, which are continuous with the Sertoli cells of ruptured spermatogenic cysts [reproduced from Nagahama (1983), with permission from Elsevier]. **(B)** Two modes of OC development [reproduced from Kerr (1919), public domain]. OCs, where ovulated oocytes are released, primarily develop in two ways: the parovarian cavity forms through the fusion of ventral ovarian tissue sheets and the coelomic epithelium, while the entovarian cavity forms through the fusion of dorso-lateral and ventro-lateral ovarian tissue sheets.

**FIGURE 6:**
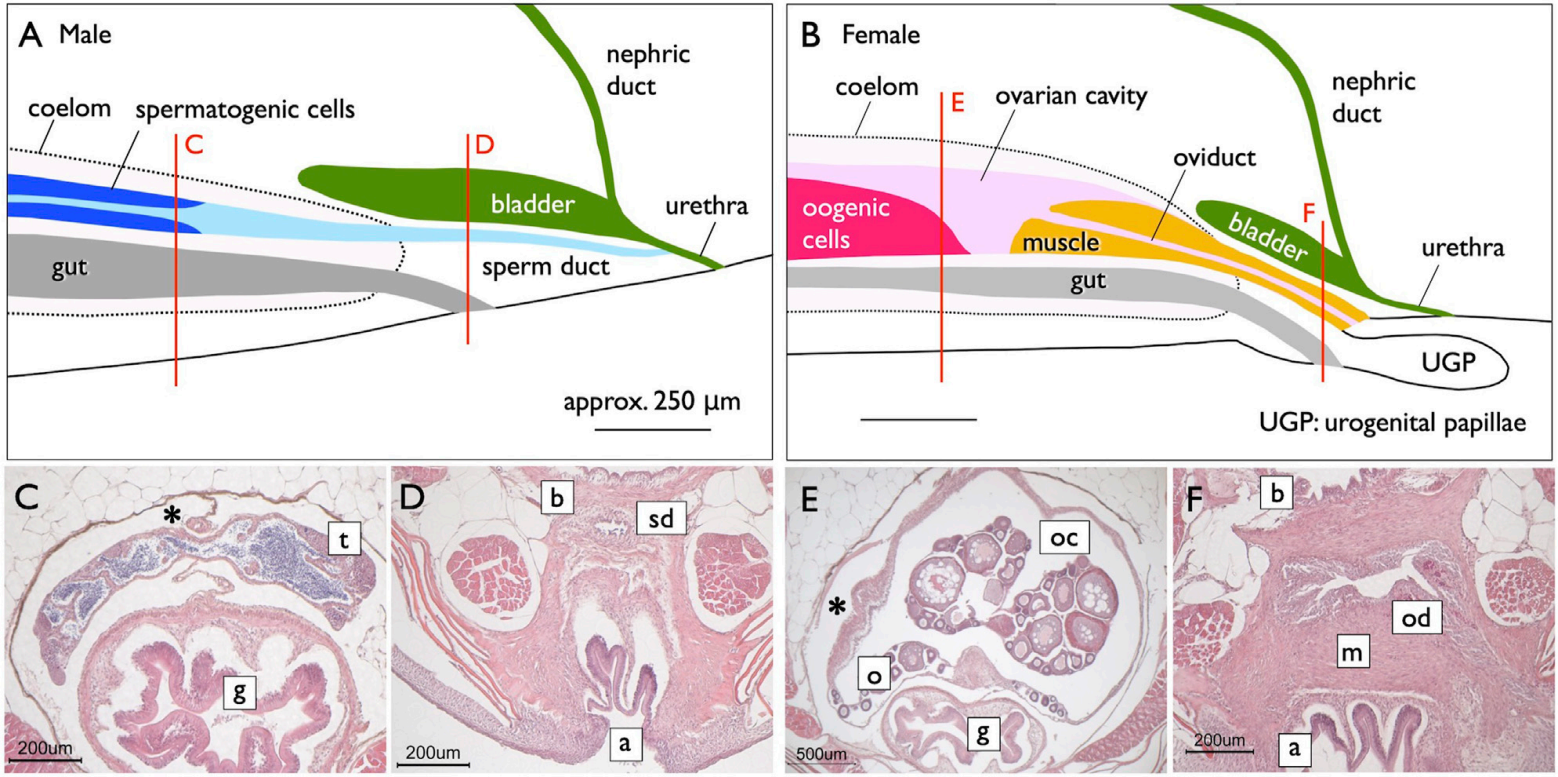
Genital ducts of the medaka [adapted from Kanamori et al. (2023), with permission from The Zoological Society of Japan]. Median plane (side view) diagrams illustrating urogenital organs in the medaka, reconstructed from serial histological sections [**(A)**, males; **(B)**, females]. The red lines indicate the levels of cross-sections shown in **(C,D)** (males) and **(E,F)** (females). In males, a single testis (t) is centrally located in the coelom (marked by asterisks) above the gut (g) **(C)**. Mature sperm are released from spermatogenic cysts and are present in canal-like spaces (light blue in A), which fuse posteriorly to form larger lumens. Further posteriorly, the lumens combine to create a single central canal (sperm duct). The sperm duct (sd) exits the coelom beneath the urinary bladder (b) and above the anus (a) **(D)**. Posteriorly, the nephric ducts join the urinary bladder (dark green in A). Subsequently, the sperm ducts fuse with the urethra from its ventral side, and the urinogenital duct opens to the exterior **(A)**. **(B)** In females, a single ovary (o) is centrally situated in the coelom above the gut (g) **(E)**. The ovarian lamella, containing oogonia and developing oocytes, is observed ventrally and at the center of the OC (oc). The posterior part of the OC lacks germ cells, and a sphincter muscle (yellow in B) protrudes from the posterior. A portion of the OC is enveloped by the sphincter muscle (m) to form the oviduct (od) posteriorly. The oviduct then exits the coelom, elongates beneath the urinary bladder **(F)**, and opens dorsally at the well-developed urogenital papillae (ugp) **(B)**.

While Nagahama (1983) provided an overview of the basic structures of genital ducts in teleosts, detailed descriptions of their developmental processes are limited, except in the medaka (Suzuki and Shibata, 2004; Kanamori et al., 2023). In male teleosts, spermatogenesis progresses within tube-like structures (Figure 5A) (Nagahama, 1983; Uribe et al., 2014). Spermatogonia are localized at the tips of tubes in tubular-type testes. In the lobular-type testes, spermatogonia are distributed at various locations along the tube. In both types of testes, once spermatogonia enter meiosis, they undergo synchronous development within cysts enveloped by Sertoli cells. As the cysts mature, their walls rupture and release mature sperm into the testicular canals. Consequently, the epithelial cells lining these canals are continuous with the Sertoli cells. Moving posteriorly, these canals merge to form a single duct that elongates further posteriorly, exits the coelom, and ultimately opens externally (Figures 6A, C, D; Suzuki and Shibata, 2004; Kanamori et al., 2023). In some species such as the medaka, the sperm duct connects to the urethra (see Dzyuba et al., 2019).

A unique reproductive structure is present in females of most non-basal teleosts (belonging to Otocephala and Euteleostei; Figure 1B). Instead of the direct release of mature oocytes into the coelom, as observed in many other jawed vertebrates, these females possess hollow ovaries. Within these ovaries, mature oocytes ovulate into enclosed spaces, referred to as ovarian cavities (OCs). OC development primarily occurs through two distinct processes: either the ventral ovarian tissue sheets elongate and merge with the coelomic epithelium, resulting in the formation of a parovarian cavity, or both the dorso-lateral and ventro-lateral ovarian tissue sheets elongate and combine to form the entovarian cavity (Figure 5B; see Lepori, 1980 for details). In the medaka, OC development is triggered by estrogen (Suzuki et al., 2004). These cavities extend posteriorly, exit the coelom, and ultimately open externally (Figures 6B, E, F; Suzuki and Shibata, 2004; Kanamori et al., 2023). In certain species, a duct connection is established with the urinary sinus (see Uematsu and Hibiya, 1983). Taken together, in teleosts, both male and female genital ducts develop similarly, involving the posterior elongation of the gonads. In males, there is no direct connection between the testes and urinary ducts (WDs) via the efferent ductules. In females, mature oocytes are not released into the coelom as in other jawed vertebrates. In this review, we denote the male and female ducts of teleosts as testicular ducts (TDs) and ovarian ducts (ODs), respectively (Figure 1A).

### 2.4 Phylogenetic distribution of each gamete-exporting organ

**FIGURE 7:**
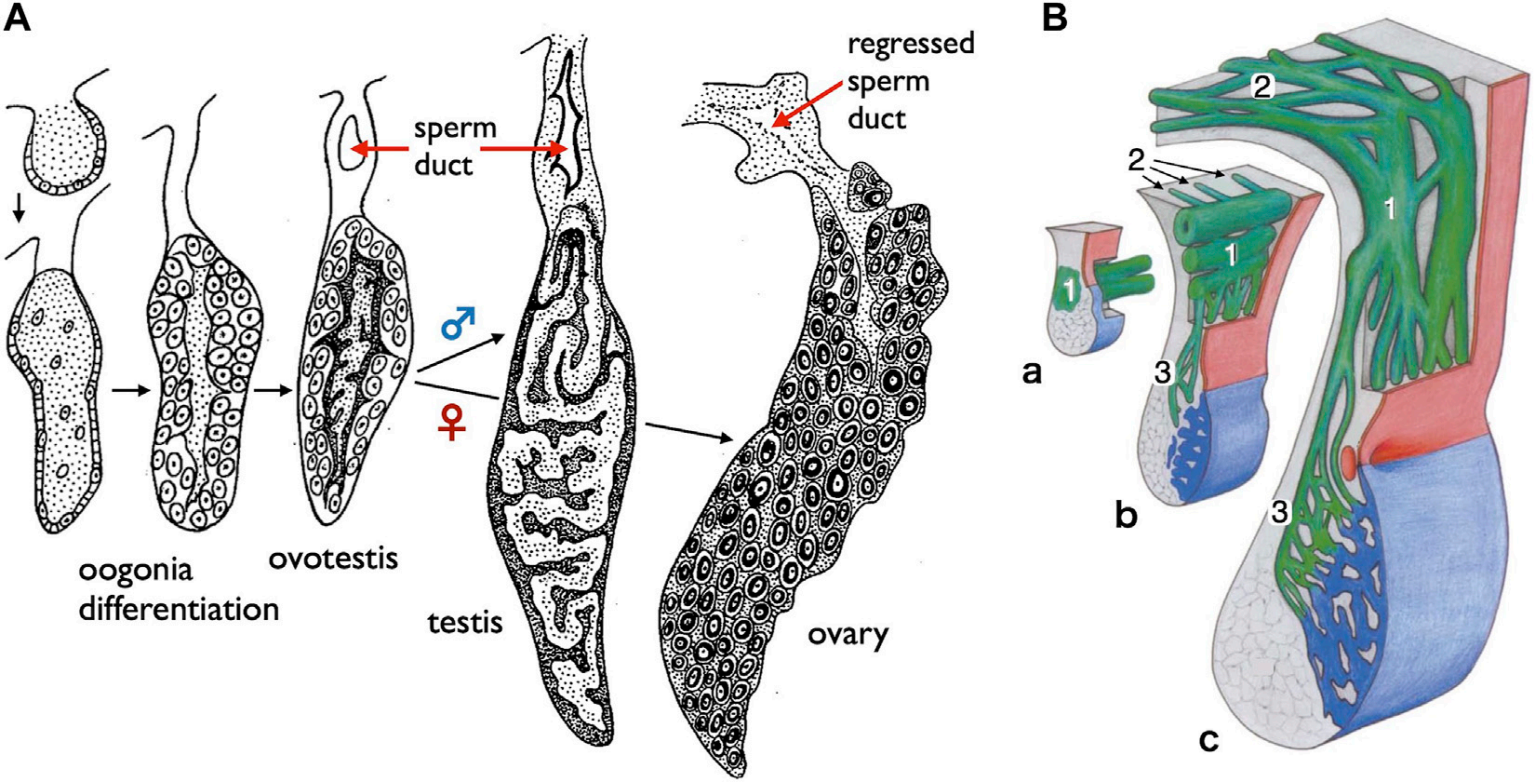
Diagrams of the sperm ducts in the European eel (a basal teleost) and the starlet sturgeon (a basal ray-finned fish). **(A)** TD development in the European eel (*Anguilla anguilla*) [adapted from Bertin (1958), with permission from Elsevier Masson SAS]. Oogonia differentiate on the surface of gonads in both sexes, followed by differentiation of spermatogenic cells in the center of gonads. At this hermaphroditic stage, the duct primordium develops dorsally to the gonad within the mesogonadium (tissue sheets suspending gonads from the dorsal wall of the coelom). After sex differentiation, the male primordia develop into the sperm ducts, while the female primordia regress. **(B)** Schematic representation illustrating the development of sperm passageways in 8–18 months-old *Acipenser ruthenus* (starlet sturgeon) [reproduced from Wrobel and Jouma (2004), with permission from Elsevier]. **a**. At 8 months, the pregonadal area of the gonadal fold contains the primary genital duct blastema (1) which grows in the caudal direction as a longitudinal system of solid anastomosing strands situated in the region of the mesogonadal attachment. **b**. At about 9 months, the primary longitudinal strands and tubules of the genital duct apparatus (1) begin to send a series of blinded tubules (2) in the direction of the opisthonephros (posterior kidney). At the same time, narrow anastomosing channels (3) traverse the mesogonadium in the direction of the gonad proper (blue). **c**. Within 12–18 months, the testicular excurrent duct system is completed. The remainders of the partly regressed primary longitudinal strands and tubules are now integrated into the transversal mesorchial ducts and represent here the portions with the largest diameters (1). The solid tubules that leave the primary longitudinal system into the direction of the opisthonephros in b have developed into the marginal longitudinal network of the kidney (2). Close to the gonad itself, the transversal mesorchial channels (3) have widened to form the longitudinal marginal network of the testis.

**FIGURE 8:**
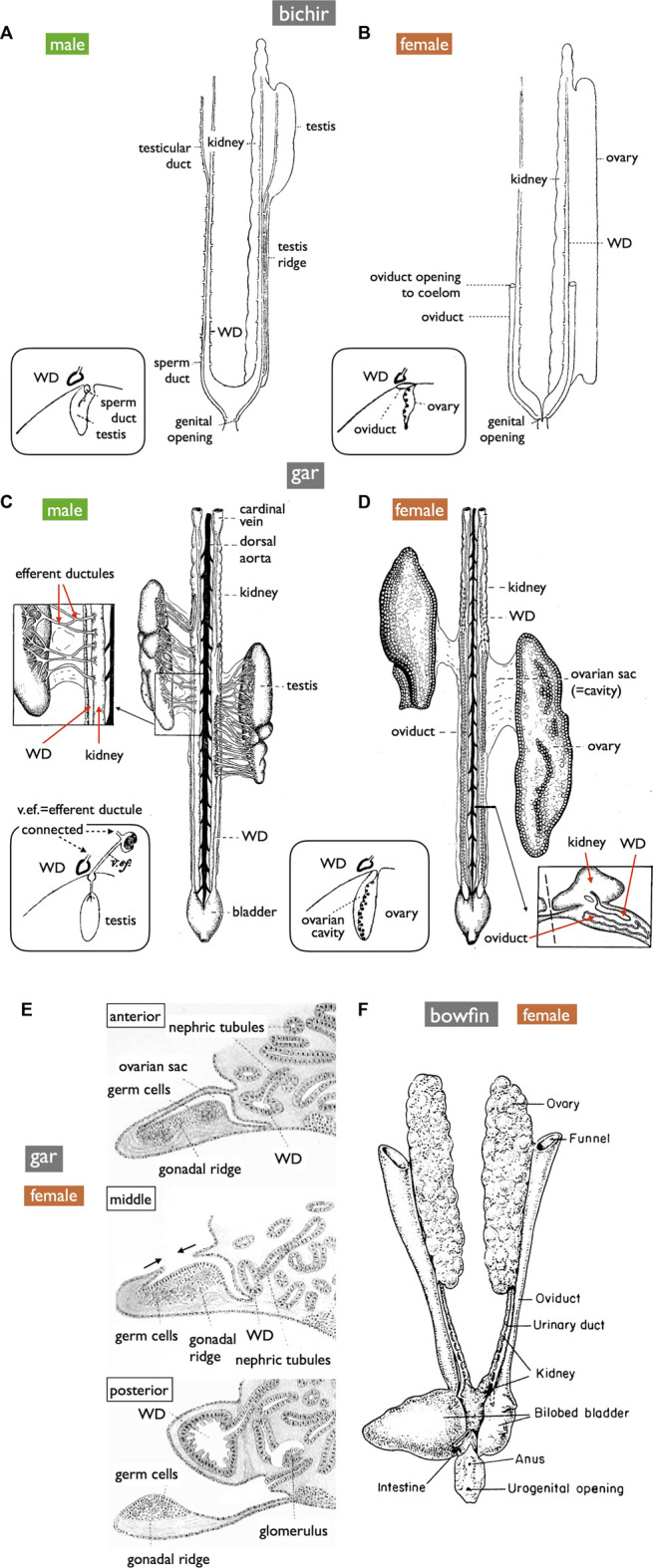
Diagrams of the urogenital systems in basal ray-finned fishes (ventral views). **(A,B)** bichir (*Polypterus*) [reproduced from Budgett (1901), with permission from The Company of Biologists]. **(C,D)** shortnose gar (*Lepisosteus platostomus*) [reproduced from Gérard (1958), after Pfeiffer (1933), with permission from Elsevier Masson SAS and John Wiley and Sons]. Insets in **(A–D)** depict cross-section diagrams from developing fishes [adapted from Budgett (1902), with permission from The Company of Biologists]. **(E)** Sketches of cross-sections illustrating OC formation in developing gar [reproduced from Balfour and Parker (1882), public domain]. **(F)** Female bowfin (*Amia*) [reproduced from Romer and Parsons (1977), with permission from Elsevier]. Male bichirs possess teleost-like sperm ducts independent of the WDs **(A)**. In contrast, male gars export sperm via thin efferent ductules, the kidney, and then the WDs **(C)**. In female bichirs and bowfins, oocytes ovulated into the coelom are exported through the openings of the MDs [**(B,F)**, respectively]. In contrast, female gars have OCs and ODs similar to teleosts **(D)**; ova are released into OCs, distinguishing them from other jawed vertebrates [inset of **(D)**]. The developmental processes of OCs resemble those of the parovarian cavities in teleosts [**(E)**, compare to Figure 5B]. OC formation appeared to progress from the anterior to the posterior direction. The anterior, middle, and posterior sections depict completed OC, OC during formation, and no OC, respecively.

Gamete-exporting organs exhibit varied distributions among vertebrates (Figure 1B). GPs are present in both sexes of cyclostomes and females of basal teleosts. It is evident that the evolution of two critical features occurred at the base of jawed vertebrate radiation: 1) the connection between the testes and WDs and 2) the *de novo* appearance of the MDs. Compelling evidence for the existence of MDs dates back to the Devonian period, approximately 380 million years ago, as indicated by the presence of intrauterine embryos in placoderm fish, an extinct group of cartilaginous fish (Long et al., 2008). Just before the diversification of teleosts, it is conceivable that male TDs evolved through the posterior elongation of the canals within the testes. Among basal teleosts, the presence of TDs has been described in the eel (Bertin, 1958) (Figure 7A). In contrast, basal teleost females have GPs, as described in Section 2.1. There has been a report of two osteoglossomorph fishes possessing OCs (Dymek et al., 2022) without descriptions of their oviducts. It is likely that at the common ancestors of Otocephala (including herrings, carps, and catfishes) and Euteleostei, teleost females acquired OCs and ODs as part of their posterior elongation. Documentation of OCs and ODs is available in Otocephala, including herring (Yamamoto, 1955), carp and goldfish (Uematsu and Hibiya, 1983), zebrafish (Hisaoka and Firlit, 1962), and catfish (Rastogi and Saxena, 1968). In Euteleostei, OCs and ODs are widespread, except in salmonids, where the OCs open secondarily into the coelom (Romer and Parsons, 1977; Lepori, 1980; Blüm, 1986; Lombardi, 1998). Two apparent exceptions to this pattern are noteworthy (Figure 1B): 1) the presence of teleost-like TDs in bichirs (Figure 8A; Budgett, 1901; Budgett, 1902) and 2) the presence of teleost-like ODs in gars (Figure 8D; Budgett, 1902; Pfeiffer, 1933). These exceptions are discussed in the following sections.

## 3 Long-unanswered questions on homologies among gamete-exporting organs in vertebrates

As mentioned earlier, most of the intriguing questions dating back to the Kerr (1919) and Goodrich (1930) eras remain unanswered. In this section, we provide a comprehensive overview of these questions.

### 3.1 Origin of GPs

In both sexes of cyclostomes and females of basal teleosts, mature gametes are released into the coelom and subsequently exported through GPs to reach the urinary sinus. Some comparative anatomists have postulated that these GPs could be homologous to the MDs in jawed vertebrates, proposing that GPs are potentially highly shortened MDs (Kerr, 1919; Goodrich, 1930). Conversely, an alternative perspective is that these pores represent primitive gamete-exporting organs, from which MDs later evolved. Topologically, both the GPs and MDs serve as paths from the coelom to the urinary sinus (Figure 1A). Another interesting hypothesis is that the GPs are homologous to the junction of MDs with the urogenital sinus in mammals. Failure to establish a connection between the MDs and urogenital sinus can lead to various developmental anomalies in the female genital tracts, including vaginal agenesis, as observed in mice (Zhao et al., 2016) and humans (Cunha et al., 2018; Parodi et al., 2022). Although the molecular mechanisms governing this connection are currently unknown, it has been suggested that retinoic acids play a role in this process (Nakajima et al., 2019). Transcriptomic analyses have been employed to study the development of the uterine-vaginal junction in birds, indicating the involvement of the TGF-beta and WNT pathways (Yang et al., 2023). Further research on the molecular and cellular aspects of GP development in cyclostomes and basal teleost females is necessary to elucidate the underlying mechanisms and evolutionary significance of these structures.

### 3.2 Testis-WD connection and evolution of teleost TDs

Anatomically, males of all jawed vertebrates, excluding teleosts and bichirs, exhibit connections extending from the testes to the WDs (Figure 1A). This urogenital connection is a characteristic feature of jawed vertebrates (Figure 4). In mammals, the rete testis is formed within the testes, and efferent ductules develop from the mesonephric kidney tubules that connect to the WDs (Shaw and Renfree, 2014; Major et al., 2021). Eventually, the rete testis and the efferent ductules are connected. Similar processes have been documented in cartilaginous fishes (see Wourms, 1977). In male gars (a basal ray-finned fish), numerous slender efferent ductules establish connections between the testes and the kidney (Figure 8C). These efferent ductules directly differentiate from the mesonephric tubules (inset of Figure 8C). However, in sturgeons, Wrobel and Jouma (2004) provided a detailed account of a distinct mode of development for the testis-WD connection. Unlike mammals and cartilaginous fishes, the primordium of this connection develops independently of both the testes and WDs (mesonephros) (1 in Figure 7B). This primordium extends its tubules towards both the testes (laterally; 3 in Figure 7B) and WDs (medially; 2 in Figure 7B) to establish a testis-WD connection. A similar duct primordium has been reported in bichirs (De Smet, 1975). The developed sperm ducts in bichirs are not connected to the WD and resemble the anatomical structure of teleost TDs (Figure 8A). It is conceivable that teleost-type TDs evolved independently in both bichirs and teleosts by bypassing tubule elongation towards the WDs from the sturgeon-like duct primordium. This evolutionary event resulted in the loss of the connection between the testes and the WDs in bichirs and teleosts. Wrobel and Jouma (2004) first proposed this hypothesis. Notably, the diagrams illustrating the TDs in the bichirs (inset of Figure 8A) and basal teleost eel (Figure 7A) demonstrate conspicuous similarities. Future research should focus on comprehensive descriptions and investigations of the molecular networks governing TD development in basal ray-finned fishes and basal teleosts.

### 3.3 Origin of teleost ODs

OC development in gars was first documented in 1882 by Balfour and Parker (Figure 8E). This process bears a striking resemblance to the parovarian cavity formation in teleosts (Figure 5B). In gars, the dorso-lateral tissue sheet elongates medially and eventually fuses with the protrusion of coelomic walls; OC formation appears to progress from the anterior to the posterior direction. In adult gars, the resulting ovarian sac or cavity (Figure 8D and inset) is connected to the oviducts and ovulated oocytes do not enter the coelom, as in other jawed vertebrates. However, in bowfins (*Amia*), a sister group of gars (see Hughes et al., 2018; Koepfli et al., 2015, for phylogeny), the oviducts anatomically resemble those of bichirs (Figure 8B) and sturgeons (Figure 10C) and can be classified as MDs (Figure 8F; Romer and Parsons, 1977). This raises questions about whether MDs were present in the common ancestors of bowfins, gars, and teleosts (Figure 1B). If so, gars and non-basal teleosts may have independently evolved OCs and ODs. If teleost-like ODs were indeed present in the common ancestors of these groups, then bowfins might have regained MDs after diverging from gars, and the GPs in basal teleost females can be interpreted as degenerated ODs, with only the pores remaining to connect to the urinary sinus.

Another hypothesis considers the possibility that MDs and ODs may be homologous organs despite their seemingly different modes of development (Sections 2.3, 3.4 below); Both MDs and ODs are lined by epithelial cells derived from the coelomic epithelium. Functional loss of orthologous genes, *Wnt4* in mice (Vainio et al., 1999) and humans (Biason-Lauber et al., 2004; Biason-Lauber et al., 2007) and *wnt4a* in zebrafish (Kossack et al., 2019) and the medaka (Kanamori et al., 2023), results in the complete absence of MDs and genital ducts, respectively. However, notable differences exist in the duct shapes and their relationships with the coelom. In mice, the anterior coelomic epithelium undergoes invagination (Figure 10A; see Section 3.4), whereas in teleosts, the genital duct primordia within the gonads elongate posteriorly and exit the coelom at the most posterior end (Figures 1A, 6B). These teleost-specific derived characters present challenges for the interpretation of homology. It is also possible that teleosts and mammals independently co-opted *wnt4a* and *Wnt4* for OD and MD development, respectively. Kanamori et al. (2023) proposed two approaches to discern correct hypotheses. The first approach involves conducting detailed molecular and cellular studies of medaka genital duct elongation to identify common genetic networks between medaka and mice, where detailed studies have already been reported (see Mullen and Behringer, 2014; Gonzales et al., 2021). The second approach adopts an evolutionary developmental biology (evo-devo) perspective as presented in the present review.

Next, we discuss the possibility of homology between teleost TDs and ODs. Notably, the zebrafish and medaka *wnt4a* mutants exhibit nearly identical phenotypes in the development of genital ducts in both sexes, suggesting similar molecular processes in the formation of TDs and ODs. The epithelium lining the lumen of TDs originates from the Sertoli cells, whereas that of ODs arises from the epithelium facing the OCs, which, in turn, is derived from the coelomic epithelium (Figure 5B). Both cell types are differentiated from the lateral plate mesoderm (Nakamura et al., 2006). However, the detailed lineages of these cells have yet to be examined in teleosts. Kanamori et al. (1985) described a group of somatic cells with a distinct basal lamina in developing medaka gonads. These cells give rise to the Sertoli cells, the epithelial cells lining the sperm duct in males, and the epithelial cells lining the OCs, and the granulosa cells in females. In mammals, Sertoli cells are derived from the coelomic epithelium (Karl and Capel, 1998).

Teleosts are the only vertebrate group that includes species capable of functional sex changes during their life cycle. A study on TDs and ODs in hermaphroditic fish species may provide insights into their possible homology. Approximately 400 hermaphroditic fish species have been reported, each exhibiting unique mechanisms of sex change (Devlin and Nagahama, 2002; Kuwamura et al., 2020). Although extensive research has focused on physiological gonadal changes during sex change, our understanding of the sexual transformation of the genital ducts remains relatively limited. The present knowledge regarding three types of sex change is summarized as follows:

#### 3.3.1 Protogynous fish (female-to-male sex change)

Female protogynous wrasse have ovaries devoid of testicular tissue. Mature oocytes are exported from the OCs through the ODs. During sex change, the ovarian tissues completely degenerate and are absorbed, resulting in the emergence of undifferentiated germ and somatic cells that subsequently transform into testicular cells. Importantly, transformed testes do not reuse the former ODs as sperm ducts. Instead, new TDs develop within the connective tissues of the ovarian surface (Hourigan et al., 1991). In some wrasse species, primary males coexist with secondary males, which are derived from females via sex change. Primary males entirely lack OCs, with only TDs observed at the base of the testes. Intriguingly, we successfully induced a reverse sex change, from male to female, in primary males by administering estrogen (Kojima et al., 2008). This induction leads to the differentiation of new OCs. Similarly, in protogynous grouper, new TDs are formed during sex change (Alam and Nakamura, 2007). Initially, these early-stage ducts appear as small, elliptical slits within the stromal tissue of the ovarian surface connective tissues. As the sex change progresses, these slit-like structures expand and fuse, eventually forming well-developed TDs.

#### 3.3.2 Protandrous fish (male-to-female sex change)

Clownfish and black porgy are protandrous hermaphroditic fish capable of transitioning from male to female. These fish exhibit bisexual gonadal structures comprising immature ovaries and mature testes during the male phase (Chang et al., 1994; Nakamura et al., 1994). As they undergo female transformation, their testes regress, facilitating ovarian development. Individuals possessing both male and female gonads maintain a unique “double-canule genital duct” structure, wherein TDs and ODs exist in the testicular and ovarian compartments, respectively (Lee et al., 2011). During male-to-female sex change in the black porgy, the male TDs regress and are replaced by connective tissue. In contrast, immature OCs and ODs develop. Although the precise changes in the genital ducts during sex change in clownfish have not been fully elucidated, the TDs no longer exist within the transformed ovary (Nakamura et al., 1994). To gain further insight into this process, we artificially induced opposite-sex changes (female-to-male) in clownfish females by administering an aromatase inhibitor (Nakamura et al., 2015). This transformation resulted in the emergence of new TDs.

#### 3.3.3 Bi-directional sex change

Okinawa rubble gobiid fish, capable of undergoing serial sex changes, possess ovaries with ODs and testes with TDs simultaneously (Kobayashi et al., 2005; Sunobe and Nakazono, 2010). Observations have shown that this goby develops genital ducts in response to changes in its social environment (Kobayashi et al., 2012).

In summary, it is widely recognized the germ and somatic cells within the gonads of hermaphroditic fish exhibit remarkable sexual plasticity. However, recent results summarized above suggest that the plasticity of genital ducts in these fish is limited and that these ducts are not reused during sex change. Instead, new genital ducts differentiate during sex change. These findings suggest that teleost TDs and ODs are independent structures that are closely associated with the physiological sex of their gonads. TDs and ODs are probably derived solely from the Sertoli cells and the epithelium of the OCs, respectively. Therefore, teleost TDs and ODs may not have homologous structures, and *wnt4a* might have been independently co-opted for their development.

### 3.4 Origin of MDs

**FIGURE 9:**
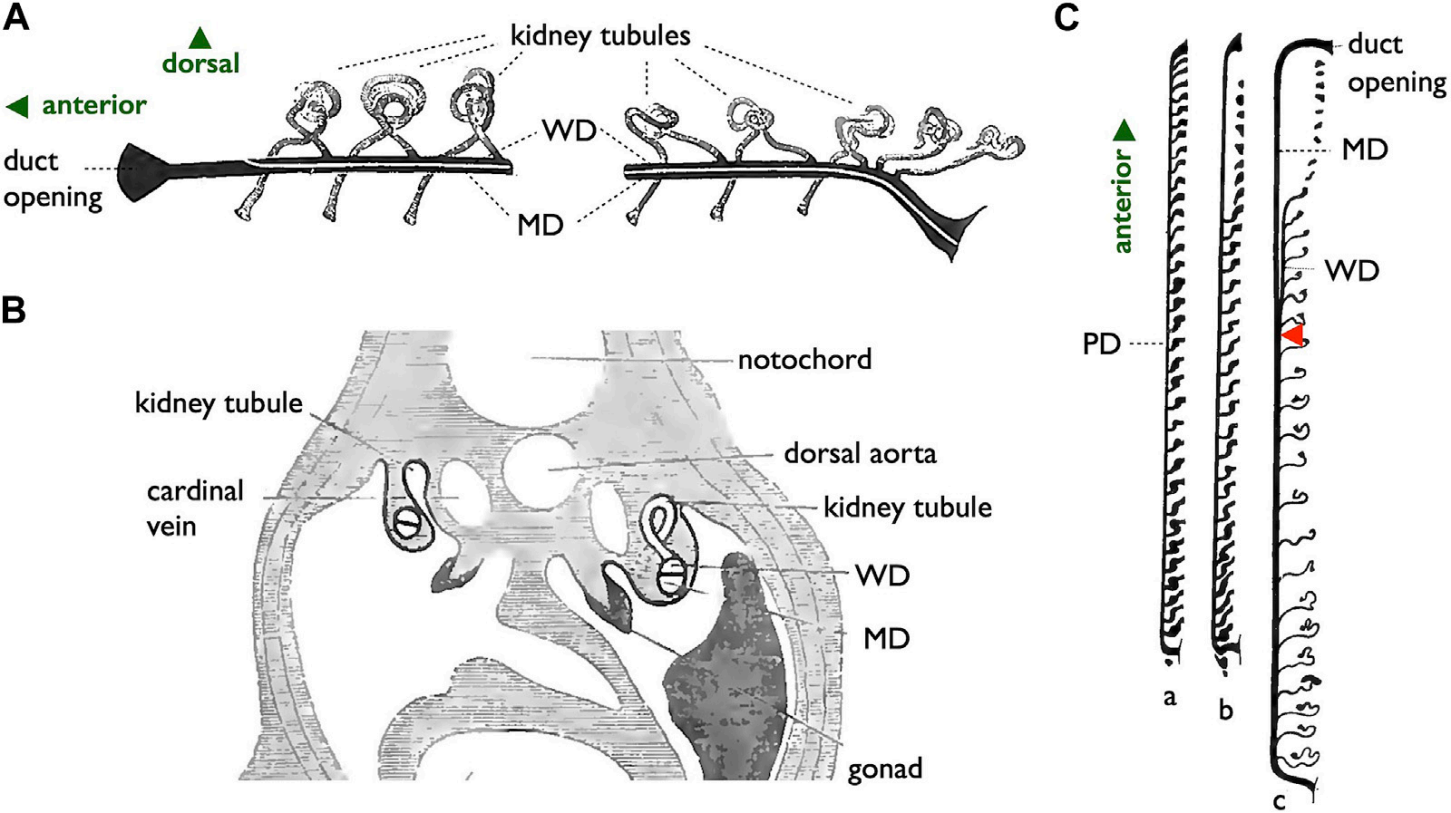
Diagrams of the **longitudinal splitting** mode of MD development in sharks. **(A,B)**, adapted from Balfour (1885), public domain. **(A)** Diagram illustrating the primitive condition of the kidney in a shark embryo. The initial pronephric duct is separated into the ventral MD and dorsal WD, which connects to mesonephric tubules. **(B)** Cross-section of a shark embryo demonstrating the WD and MD formation by the **longitudinal splitting** of the pronephric duct. **(C)** Arrangement of the pronephric duct, etc., in embryos of a shark, *Pristiurus*. a, male 17 mm. Pronephros are connected to the pronephric duct (PD). b, female 19 mm. The anterior pronephros degenerated. c, female 27 mm. The PD is split into the ventral MD and dorsal WD. The point of splitting is labeled with a red arrowhead. Presumably, this splitting point moves posteriorly as development proceeds [adapted from Kerr (1919), after Rabl (1896), public domain].

The challenge at hand is to understand the existence of two distinct modes of MD development, a topic that ignited extensive debate among comparative anatomists around the turn of the 20th century (Kerr, 1919; Goodrich, 1930). Wourms (1977) provided a comprehensive summary of MD development in cartilaginous fishes as follows: “Müllerian ducts develop from the pronephros and the pronephric duct. . .The main portion of the Müllerian duct develops from the pronephric duct. The pronephric duct undergoes a gradual **longitudinal splitting** into an anterior-posterior direction to produce a dorsal and ventral tube. The ventral tube is continuous with the pronephric funnel [opening to the coelom] and becomes the Müllerian duct. The dorsal tube receives the kidney tubules. It is a true Wolffian (mesonephric) duct which persists as the functional urinary duct of the opisthonephros. . . . (Kerr, 1919; Goodrich, 1930).” These descriptions were sourced from two classical textbooks and are rooted in original papers from the late 19th century, contributed by notable authors such as Semper (1875), Balfour (1875), and Rabl (1896). Figure 9 shows several original diagrams from these pioneering studies. Importantly, Figure 9C reveals a connection between the posterior kidney tubules and pronephric ducts before the splitting process, a feature not observed in red stingrays or catsharks (Figures 12, 13, as discussed below).

**FIGURE 10:**
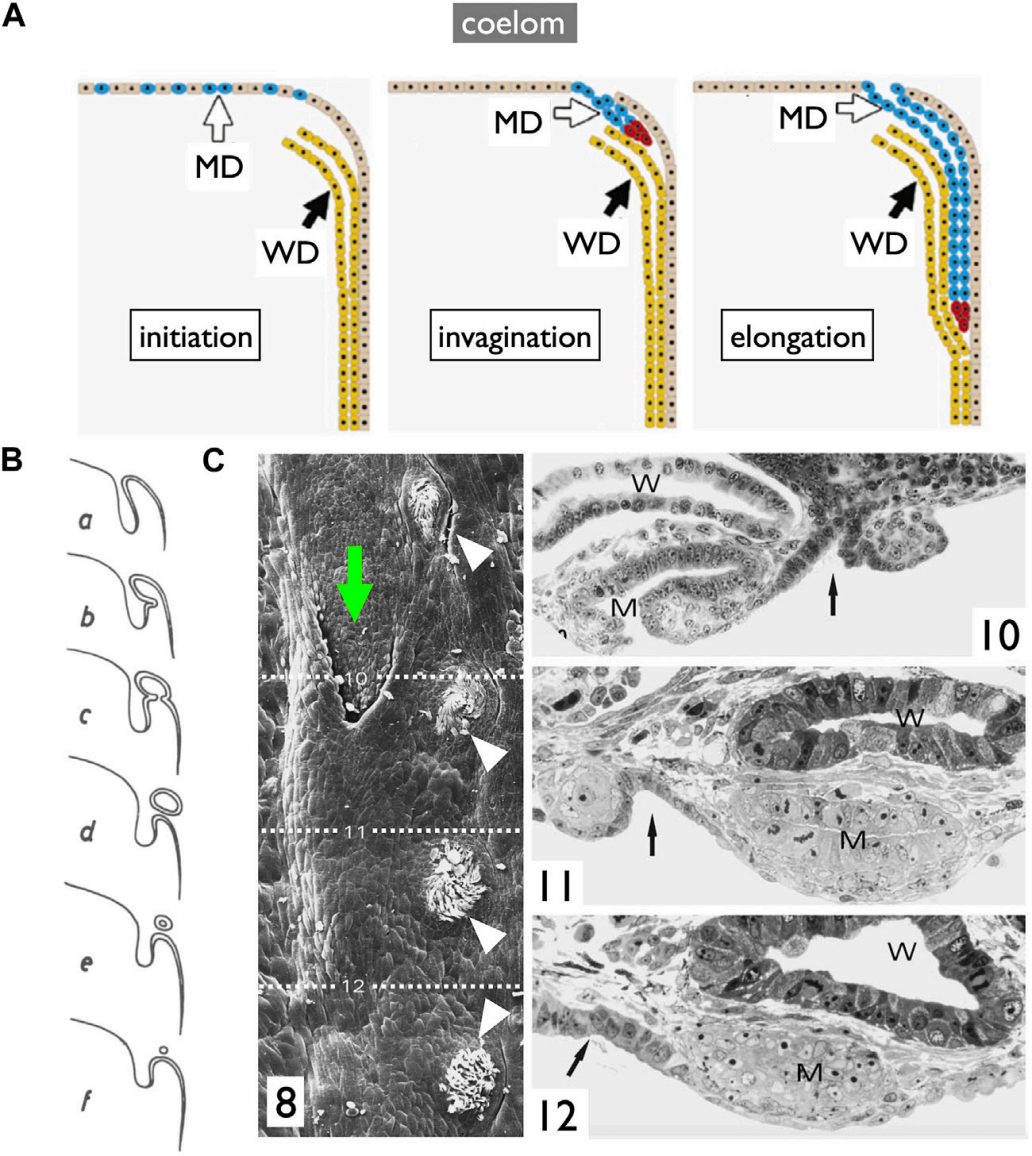
Diagrams of the **invagination-elongation** mode of MD development. **(A)** A three-phase model for MD development in mammals. In the first phase (initiation), cells of the coelomic epithelium are specified to become MD cells (blue). After specification, the second phase (invagination) begins and these cells invaginate posteriorly towards the WD. Once the MD comes into contact with the WD, the third phase (elongation) begins and the MD elongates posteriorly, following the WD path, towards the urogenital sinus. Red cells; proliferating MD precursor cells, brown cells; coelomic epithelial cells, yellow cells; WD epithelial cells [reproduced from Orvis and Behringer (2007), with permission from Elsevier]. **(B)** Oviduct development process in bichirs [reproduced from De Smet (1975) with a permission from KMDA] shown in cross-sections from anterior to posterior direction (a–f). Anteriorly, the coelomic epithelium thickens with cuboidal cells (a). At the dorsolateral base of the genital ridge, they undergo invagination (b,c), forming a posteriorly-extending tubular structure (d,e) that ultimately closes farther posteriorly (f). **(C)** Oviduct development in sturgeons [adapted from Wrobel (2003), with permission from Springer Nature]. 8, 28-day-old *Acipenser ruthenus*, SEM. A series of segmentally arranged nephrostomes (coelomic openings of nephrons; white arrowheads) coexists with an opening of the MD (green arrow). The numbers 10–12 with dotted lines represent the locations of cross-sections similar to those in 10C-10, 11, 12: 10, the level of the opening of MD (left body side); 11, the primordium of MD with a slit-like lumen (right body side); 12, the posteriorly growing solid tip of MD primordium (right body side). Arrow, a line of nephrostomes; W, WD; M, MD.

**FIGURE 11:**
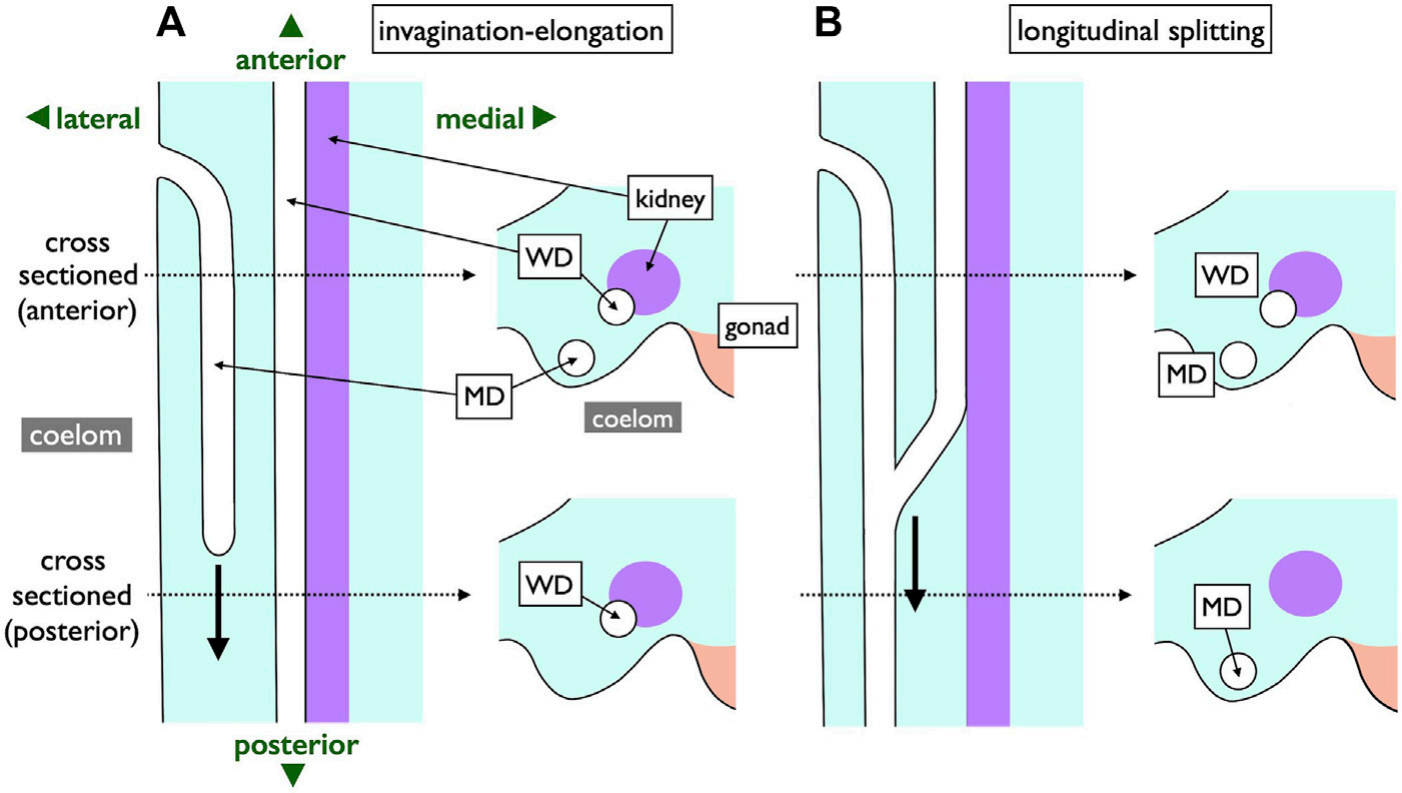
Comparison of two MD development modes depicted with ventral views (left) and transverse views (right). Two dotted lines represent levels of cross-sections. In the **invagination-elongation** mode **(A)**, the MD epithelium invaginates and elongates posteriorly (thick arrow). In contrast, the pronephric duct splits into the ventral MD and dorsal WD in the **longitudinal splitting** mode **(B)**. The splitting point moves posteriorly (thick arrow) as development proceeds. The only difference is the location of a duct in posterior sections; WD is proximal, and MD is distal to the kidney within nephric ridges.

In contrast, an alternative mode of MD development is prevalent among most jawed vertebrates (Figure 10). During this process, the anterior coelomic epithelium near the pronephros undergoes thickening and invagination. The tip of this invaginated cord of cells elongates posteriorly along the WD until it reaches the urinary sinus. This mode, **invagination-elongation**, has been described in various species, including bichirs (Budgett, 1902; De Smet, 1975), sturgeons (Wrobel, 2003), amphibians (Hall, 1904; Wrobel and Süß, 2000), birds (Gruenwald, 1941; Jacob et al., 1999), and mammals (Gruenwald, 1941) (Figure 1B). The process has been most comprehensively documented in mice, including detailed cellular and molecular dynamics (Figure 10A; Orvis and Behringer, 2007; Mullen and Behringer, 2014; Gonzales et al., 2021). In mice, specific coelomic epithelial cells have been identified as the MD epithelium through the expression of several transcription factors such as LHX1, PAX2, and EMX2. These cells subsequently undergo invagination and posterior elongation under the influence of WNT4, which is expressed in the mesenchymal tissue underlying the tips of the elongating MDs. This mode is also highly likely to be applied in bichirs, as indicated by developmental diagrams of the oviducts (Figure 10B; De Smet, 1975). The coelomic epithelium at the dorsolateral base of the genital ridge undergoes invagination (b–c), leading to the formation of a posteriorly extending tubular structure (d–e), which ultimately closes further posteriorly (f). Unfortunately, older specimens that the author did not obtain may reveal an MD connection to the urinary sinus. The development of MDs in sturgeons has been meticulously studied (Figure 10C; Wrobel, 2003). Scanning electron microscopy (SEM) and cross-section images unequivocally illustrate that sturgeon MDs develop in a manner very similar to that observed in mice, involving invagination of the coelomic epithelium (10C-8, 10), followed by posterior elongation (10C-8, 11, 12). In conclusion, the gradual **longitudinal splitting** during MD development is currently exclusive to cartilaginous fishes. Although this mode was previously described in urodeles (Furbringer, 1878; Romer and Parsons, 1977), it has since faced considerable skepticism (Hall, 1904; Wrobel and Süß, 2000). Distinguishing these two modes based solely on histological sections poses a significant challenge, as shown in Figure 11. In both cases, the anterior cross-sections exhibited striking similarities, with the primary discerning factor residing in the position of the ducts in the posterior cross-sections. This distinction may be difficult to verify, with WDs positioned proximally and MDs positioned distal to the kidney.

**FIGURE 12:**
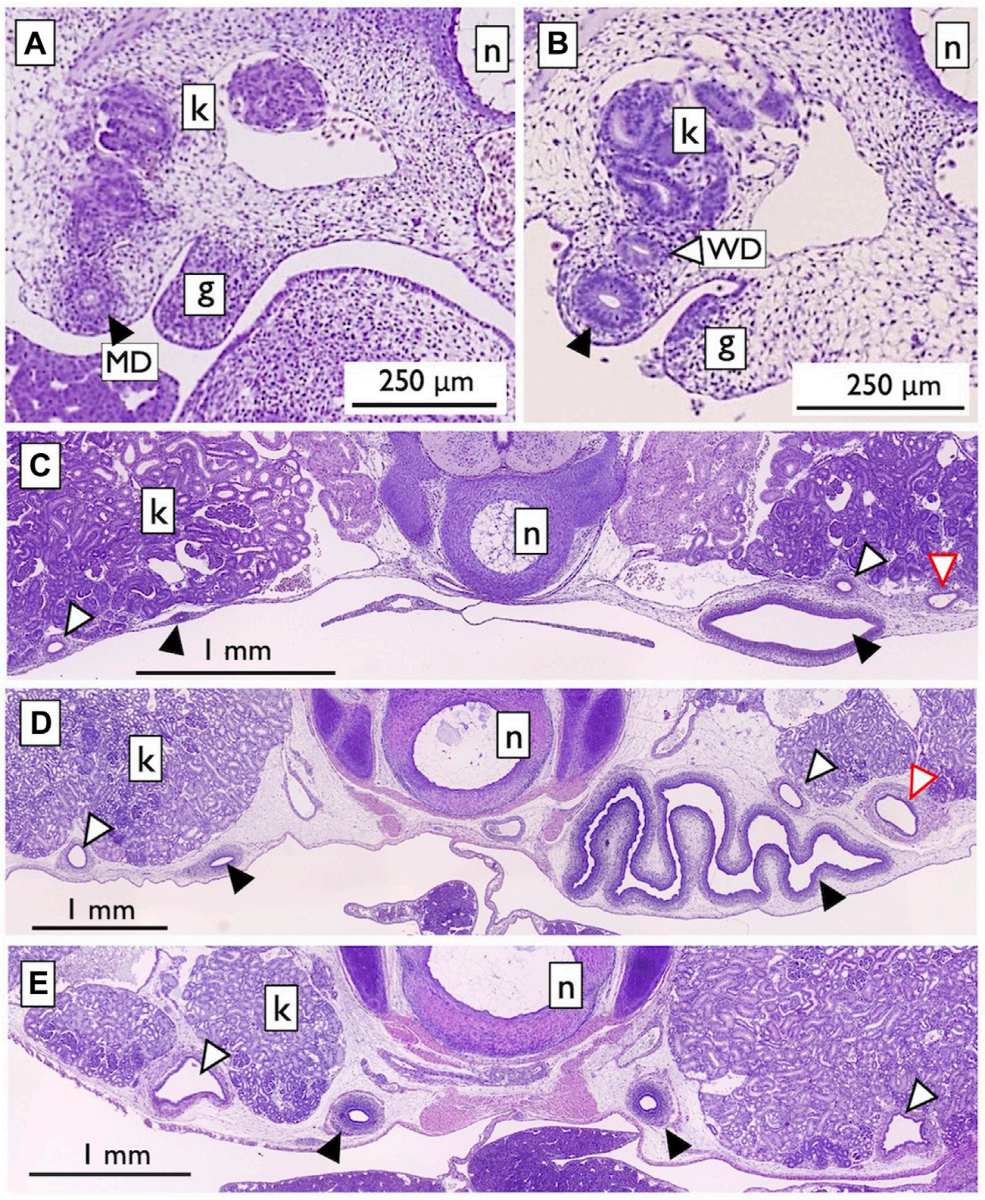
Urogenital duct development in the viviparous red stingray fetuses [adapted from Kobayashi et al. (2021), with permission from The Japanese Society of Fisheries Science]. **(A)** Fetus with a total length (TL) of 1 cm. The nephric ridge is shown adjacent to the gonad (g). The kidney (k) was still in the developmental stage, with no associated duct. In contrast, the completed pronephric duct was located distally in the nephric ridge (labeled as MD; closed arrowheads). **(B)** Fetus with a TL of 2 cm. The WD (open arrowheads) was observed near the kidney. **(C)** Female fetus (TL 8.2 cm). The MDs on the right side were enlarged and differentiated into the uterus. No changes were observed in the MDs on the left side, as well as in the left and right WDs. Secondary nephric ducts (red open arrowheads) were also observed. **(D)** Female fetus (TL 10.1 cm). Myometrium muscle development was observed. **(E)** Male fetus (TL 10.3 cm). Slight enlargement of the left and right WDs was observed. n; notochord.

**FIGURE 13:**
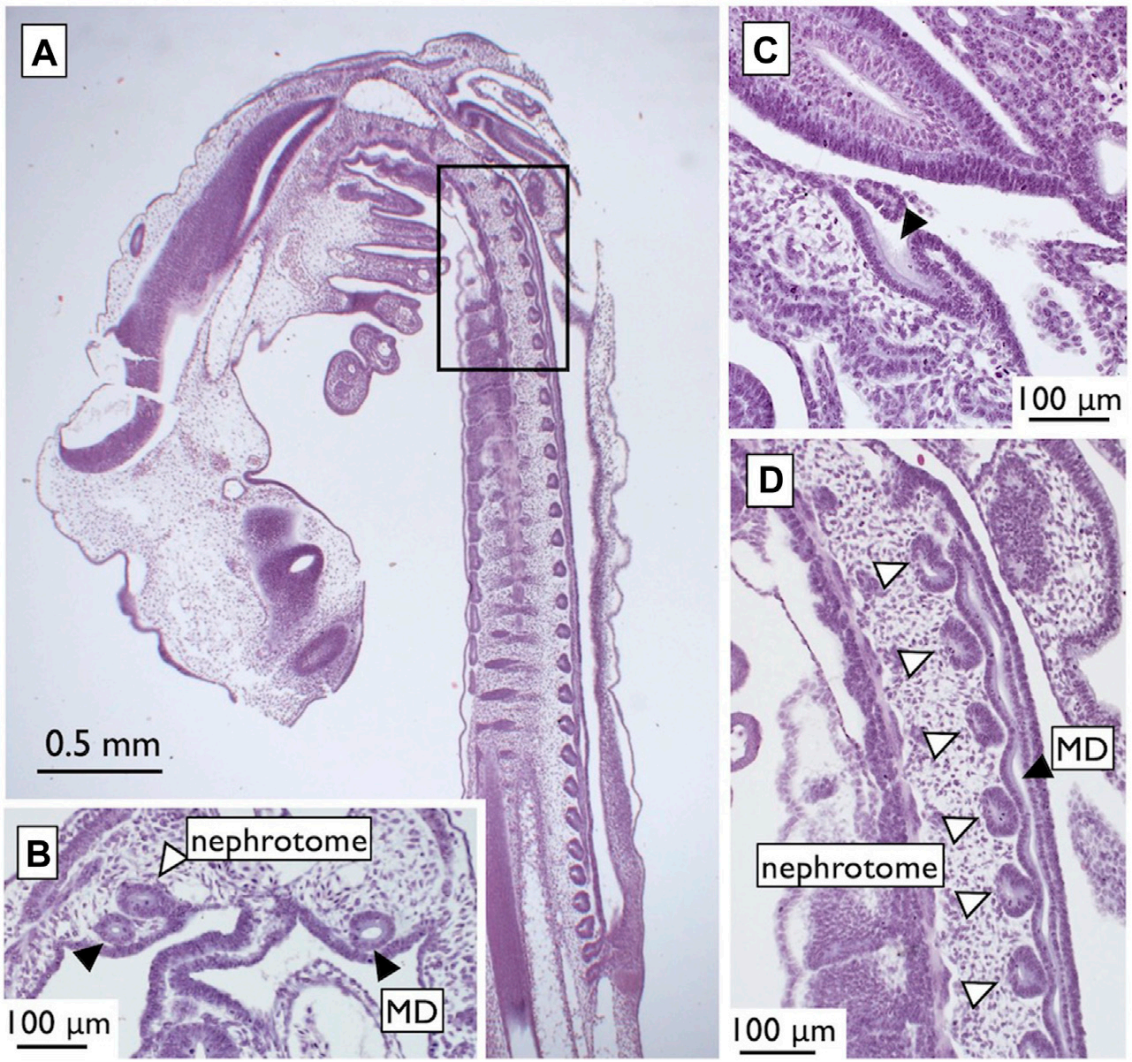
Urogenital systems in the catshark embryos at 45 days after spawning [adapted from Kobayashi et al. (2021), with permission from The Japanese Society of Fisheries Science]. **(A)** A low-magnified micrograph of a parasagittal-section. A blow-up of the squared portion is shown in **(D)**. The MDs (closed arrowheads) were formed, but the mesonephric kidney was still undifferentiated and present as segmental nephrotomes (open arrowheads). In a cross-section shown in **(B)**, the MDs were observed in the ventral nephric ridge distal to the nephrotome. In a parasagittal-section near **(A)**, the opening of MD to the coelom was observed [**(C)**, arrowheads].

Therefore, we reexamined the histological slides originally prepared by one of the authors (YK; Kobayashi et al., 2021). In viviparous red stingrays (*Hemitrygon akajei*), we observed the differentiation of MDs on the ventral side of the kidney primordium in immature fetuses, approximately 1 cm in total length, where gill formation was completed (Figure 12A). When the fetuses reached a length of 2 cm and placental development was initiated, WDs became discernible (Figure 12B). In female fetuses measuring approximately 8 cm in body length, the right MD enlarged and differentiated into the uterus (Figure 12C). In the subsequent developmental stages, we observed growth of the inner uterine wall and myometrium in female fetuses just before birth, at a body length of approximately 10 cm (Figure 12D). Therefore, it is evident that the left-right asymmetry of the genital ducts observed in adult females was already established during intrauterine development. In contrast, we observed only a slight enlargement of both the left and right WDs in male fetuses immediately before birth (Figure 12E). WD differentiation into sperm ducts and seminal vesicles is expected to occur after birth. Importantly, the MDs in male fetuses and WDs in female fetuses did not regress; instead, they persisted as undifferentiated ducts. Besides studies on stingrays, we conducted a comprehensive analysis of the differentiation and developmental processes of genital ducts in the oviparous catshark (*Scyliorhinus torazame*). In embryos examined 45 days after spawning, a pair of left and right MDs originating from the former pronephric ducts were observed on the dorsal side of the body cavity (Figure 13). The most anterior part of the MDs opened into the coelom (Figure 13C). At this stage, the mesonephric kidney had not yet undergone differentiation, and only segmental development of the nephrotomes was observed (Figures 13A, B, D). This observation demonstrates that MD differentiation occurs before mesonephric kidney or WD differentiation in catsharks as well. Subsequently, in female embryos, the anterior part of the MDs undergoes enlargement and differentiation into eggshell glands (Kobayashi et al., 2021). Similar to the stingrays, the WDs in females and the MDs in males persisted without regression, remaining as undifferentiated ducts (Kobayashi et al., 2021). In both species, as reported in previous studies by Balfour (1875), Semper (1875), and Rabl (1896), the pronephric ducts differentiated long before the onset of mesonephros development. During this early stage, the nephrotomes remained undifferentiated, whereas well-developed pronephric ducts (referred to as MDs in our definition, as outlined in Section 2.2) were present (Figure 12, 13). Based on our observations, the pronephric ducts are directly differentiated into the MDs. This inference is primarily based on the spatial arrangement of these ducts, with the MDs consistently situated distally in the nephric ridge, compared to the dorsal and proximal location of the WDs (Figure 12). Thus, the results from the late 19th century were partially confirmed. However, we were unable to identify evidence of pronephric duct splitting in either the red stingrays or the catsharks. Furthermore, the differentiation of WDs in these species and the establishment of their connections to the testes remain elusive. This uncertainty is likely due to the absence of crucial samples in the developmental series examined.

In summary, the confusion persists as Goodrich noted in 1930: “Obviously, there is a striking difference between the development of the Müllerian ducts in Selachii [sharks and rays] and Tetrapoda; indeed, many have doubted its homology in the two groups. Yet so similar are the ducts in the adult condition both in function and in anatomical relationship that it can scarcely be doubted that they are homologous throughout the Gnathostomes [jawed vertebrates] (leaving the Teleostomes [teleosts] aside for the present. . .). . . . Further knowledge of the development in other groups may enable us to solve this problem.” Future research focusing on MD development in cartilaginous fishes, such as sharks and rays, is of utmost importance. Wrobel (2003) similarly argued as follows: “In *Acipenser* [a sturgeon], the müllerian ducts unite with the corresponding wolffian ducts before the latter fuse to form the urogenital sinus. Without knowing its embryogenesis, the fully-developed adult condition can easily be misinterpreted as the result of an incomplete longitudinal splitting of the wolffian ducts. This diagnostic error was obviously made by Romer (1960) [see Romer and Parsons (1977)] who considered the müllerian duct of sturgeons to be a side branch of the opisthonephric duct and the starting point of a morphogenetic line leading to the situation seen in elasmobranchs [sharks and rays] and urodeles. In these two groups, the müllerian duct allegedly is the result of a complete longitudinal splitting of the wolffian duct, an assumption, however, that has been proven untenable for the lower amphibians (Hall, 1904; Wrobel and Süß, 2000) and certainly needs reinvestigation with a suitable methodology for the elasmobranchs.”

Finally, we briefly explore the possibility that WDs and MDs are homologous organs. Romer and Parsons (1977) expressed this perspective as follows: “The embryonic course of the oviduct [MD] parallels that of the archinephric duct [WD]…. The evidence on the whole thus indicates that the female genital duct (like that of the male. . .) is derived from the urinary system but has become so specialized that embryonic evidence of its origin may be lost.” Interestingly, quite a few genes are expressed in either the epithelium or mesenchyme of both MDs and WDs during development (Mullen and Behringer, 2014; Gonzales et al., 2021). This shared expression pattern raises the possibility of a deep homology between MDs and WDs.

In conclusion, we still lack detailed temporal sequences of urogenital development in cartilaginous fishes for comparison with those in mammals.

## 4 Prospects and proposals

As emphasized throughout this review, a significant knowledge gap persists concerning the fundamental structures and developmental processes of gamete-exporting organs in essential vertebrate groups such as cyclostomes, cartilaginous fishes, basal ray-finned fishes, and basal teleosts. Several factors have contributed to the limited research in this area. Obtaining a sufficient number of samples for analysis poses a significant challenge because of the rarity of many of these species and the difficulties in maintaining them in laboratory settings. Historical studies conducted around the turn of the 20th century have often relied on small sample sizes, further complicating our understanding. Moreover, the relatively late genital duct differentiation during development, which sometimes occurs several years after fertilization, poses two distinct challenges. First, determining the precise timing for sampling to analyze duct development is challenging, as the variability in developmental timing increases with prolonged development. Secondly, larger body sizes at the time of sampling create difficulties in histological sectioning, requiring meticulous trimming, a greater number of sections, and more resources.

Given these challenges, we propose three approaches to advance the study of duct structure and development.

### 4.1 MRI application on living specimens

Observing organ structures in living organisms without sacrificing them would help to overcome the limitations of sample availability. Researchers can utilize the same organism at various developmental time points. Multiple noninvasive imaging techniques, including computed tomography (CT), magnetic resonance imaging (MRI), positron emission tomography (PET), single-photon emission computed tomography (SPECT), and ultrasound (US), are available (see https://encyclopedia.pub/entry/12494; Riehakainen et al., 2021). It is preferable to avoid the use of radioactive tracers or heavy molecular dyes. Therefore, MRI is a promising option among these methods. MRI has been successfully utilized in humans to detect developmental anomalies of MDs owing to its exceptional soft tissue resolution and accuracy (Udayakumar et al., 2023). Moreover, MRI has exhibited outstanding resolution, nearly equivalent to histology, for the imaging of mouse embryos (Turnbull and Mori, 2007). Recent studies have indicated that MRI resolution is sufficient for observing gamete-exporting organs in vertebrates (e.g., Kanahashi et al., 2023). Fortunately, the species we intended to analyse, such as lampreys, sharks, rays, bichirs, bowfins, sturgeons, gars, and basal teleosts such as eels, are sufficiently large to be accommodated in readily available MRI facilities. The living yellowtail specimens (rather large fish, 60–70 cm body length) have been imaged by MRI successfully (Tawara et al., 2022). Observing organ structures *in situ* without sacrificing specimens allows researchers to determine the ideal time for sacrifice and subsequent organ dissection for traditional histological analyses. Nonetheless, challenges may arise due to the accessibility of MRI facilities and the associated expenses for imaging animal specimens.

### 4.2 Genetic and cellular atlas/dynamics during development of gamete-exporting organs

Recent advances in single-cell RNA sequencing technologies, combined with techniques for mapping cell types in 3D spatial contexts (spatial transcriptomics; see Rao et al., 2021; Moses and Pachter, 2022; Williams et al., 2022; Tian et al., 2023), offer valuable methods for investigating limited sample populations. With prior knowledge of the timing and locations for sampling from non-invasive imaging, researchers can confidently proceed with spatial transcriptomic analyses. These analyses can potentially reveal the genetic networks involved in various cell types during gamete-exporting organ development in vertebrates. By comparing cell types and networks, we can gain insight into the presence or absence of homology among these organs.

### 4.3 Mutants of *wnt4a* and other genes involved in MD development

One of the most practical approaches currently available involves CRISPR/Cas9-mediated gene knockout of *Wnt4* (*wnt4a*) or other genes implicated in mammalian MD development (Mullen and Behringer, 2014; Gonzales et al., 2021). Researchers can investigate the possible phenotypes arising in gamete-exporting organs following gene knockouts. Importantly, CRISPR/Cas9 systems can theoretically be applied to most organisms, making them suitable for genetic research (see Russell et al., 2017; Matthews and Vosshall, 2020). Notably, CRISPR/Cas9-mediated gene knockout has already been reported in the lamprey, one of our target organisms (Suzuki et al., 2021). Nevertheless, a significant challenge arises due to the relatively long developmental timeline of gamete-exporting organs in the proposed organisms. Successful husbandry methods are required to maintain these animals in laboratories or aquaria for extended periods. In this context, noninvasive imaging methods are indispensable for determining the optimal time to sample duct development in mutants, which is likely to vary among individuals.

## 5 Concluding remarks

The presence of gamete-exporting organs is vital for the reproductive success and survival of vertebrates. Based on the phylogenetic distribution of these organs (Figure 1B), it is likely that several transitions between the organ types occurred during evolution. For example, there were changes from cyclostome GPs to WDs and MDs in cartilaginous fishes, as well as transitions from WDs and MDs in basal ray-finned fishes to teleost TDs and ODs. How were these evolutionary changes successfully implemented? Are the MDs of sharks and rays truly homologous to those of other jawed vertebrates? If not, what kind of independent genetic networks underlie the development of seemingly identical organs in adults? These questions remain unanswered after a century of limited scientific research. We firmly believe that these challenges are worth pursuing and may be addressed with modern technologies that were unavailable around the turn of the 20th century when the subject was ever so popular.

## Abbreviations

WD, Wolffian duct, MD, Müllerian duct, GP, genital pore, TD, testicular duct, OD, ovarian duct, OC, ovarian cavity.
